# Management goals of type 1 Gaucher disease in South Africa: An expert Delphi consensus document on good clinical practice

**DOI:** 10.1371/journal.pone.0290401

**Published:** 2023-08-22

**Authors:** Vernon Johan Louw, Ilanca Fraser, Pilar Giraldo

**Affiliations:** 1 Department of Medicine, Division Clinical Haematology, Groote Schuur Hospital, University of Cape Town, Cape Town, South Africa; 2 VI Research (Pty) Ltd, Centurion, South Africa; 3 Fundación Española Estudio y Terapéutica Enfermedad de Gaucher y otras Lisosomales (FEETEG), Zaragoza, Spain; Bay Area Hospital, North Bend Medical Center, UNITED STATES

## Abstract

**Background:**

Gaucher disease is a rare autosomal recessive glycosphingolipid storage disease that ultimately leads to reduced life expectancy. Management of Gaucher disease is challenging due to its wide genotypic and phenotypic variability and changing clinical manifestations due to effective treatment. Deliberation between experts is essential to discuss daily clinical practice and identify controversies regarding the management of Gaucher disease. The usefulness of methods like Delphi surveys is suitable for setting up consensus recommendations for different clinical scenarios.

**Objectives:**

The goal of this study was to develop an expert consensus document for the management of type 1 Gaucher disease by local experts.

**Methods:**

A modified e-Delphi was carried out to develop an expert consensus document on the management goals of type 1 Gaucher disease in South Africa. Following a literature review and input from the steering committee, 205 management goals and best practice statements were e-mailed to an independent panel for consensus development using three rounds of voting. The panel consisted of five local healthcare practitioners with expertise in Gaucher disease. Each panelist provided independent evaluations of statements sent to them via a dedicated survey platform. Panelists indicated their level of agreement on a 9-point Likert scale (1 = absolute disagreement to 9 = absolute agreement) during each round of voting. The criteria to retain a statement in the final round were ≥80% high agreement (7–9).

**Results:**

193 statements met the consensus threshold after three rounds of voting and were included in the final guidance document. In general, the management goals presented in this paper are in line with existing literature on the subject. Additional management goals and general recommendations on sound clinical practice, obtained from more recent research and the panelists’ own clinical experience, have been included to develop a comprehensive consensus document on the management goals of type 1 Gaucher disease.

**Conclusion:**

This paper provides high-level guidance with respect to management goals, and the use of current therapies and adjunctive interventions in type 1 Gaucher disease to assist clinicians in their decisions about the appropriate management of patients in everyday clinical practice. These management goals and best practice statements might be used to inform an update to future South African guidelines on the disease.

## Introduction

Gaucher disease (GD), a rare autosomal recessive glycosphingolipid storage disease, is the most common lysosomal storage disorder (LSD), with an estimated worldwide prevalence of 1 in 50,000–100,000 in the general population. and ∼1 per 850 in the Ashkenazi Jewish population [1–3]. In South Africa, GD has been demonstrated to occur in all ethnic groups [4].

GD is divided into 3 clinical types: type 1 (adult type), type 2 (infantile type or acute neuropathic), and type 3 (juvenile, subacute, or chronic neuronopathic) [5]. GD is caused by an enzyme (glucocerebrosidase) deficiency that leads to the storage of complex lipids in certain types of blood cells, which creates glycosphingolipid-laden macrophages (Gaucher cells) throughout the liver, spleen, bone marrow, skeleton, and occasionally the lungs [6]. Type 1 Gaucher disease (GD1) (OMIM # 230800) is the most common type of GD and accounts for more than 90% of all GD patients [6, 7]. Defined as the non-neuronopathic subclass, GD1 is a chronic multi-organ disorder without neurological involvement, which can present at any age [8]. GD types 2 and 3 are known as neuronopathic GD as both types have early onset neurological involvement that progresses over time. Type 2 GD presents in infancy and is fatal, usually before the age of two years. Type 3 GD exhibits the visceral manifestations described in GD1, usually combined with oculomotor neurological involvement. Type 3 GD typically presents in early childhood and has a more gradual progression [5].

GD1 presents the broadest phenotypic spectrum in GD with respect to the age of onset, rate of progression, and organs affected [1]. The severity and clinical course of the disease are also extremely heterogeneous, varying between patients and affected domains in individual patients [9]. Patients with GD1 display a variety of symptoms, ranging from those with child-onset to asymptomatic disease [10]. Anemia, thrombocytopenia with frequent bleeding, enlargement of liver and/or spleen (hepatosplenomegaly), and skeletal abnormalities—including osteopenia with bone pain, pathologic fractures, osteonecrosis, osteoporosis, lytic lesions, bone crises and/or infarcts, joint avascular necrosis, bone marrow infiltration with bone medullary and skeletal deformities—are common clinical manifestations of GD1 [2, 3, 9–11]. Lung involvement with interstitial lung disease and pulmonary hypertension [10], cholelithiasis, and autoimmune phenomena may occur in a small number of GD patients [3].

The diagnosis of GD is based on history, clinical evaluation, laboratory investigations, and diagnostic imaging. Guidelines for diagnosing GD in the South African setting have been covered elsewhere [4] and are beyond the scope of this study. However, it is important to note that early diagnosis is imperative for the timely initiation of specific therapy before the development of irreversible complications, and for prenatal diagnosis in subsequent pregnancies [2]. Once a diagnosis of GD is confirmed, a comprehensive evaluation of all clinically relevant disease domains is imperative for the development of personalized therapeutic goals and effective monitoring of these patients [1, 10].

Management of GD requires a multidisciplinary approach and is best coordinated at a center with expertise in this complex disease, managed by a multidisciplinary team (MDT) adept at recognizing the diverse clinical manifestations and managing potential GD-related or -unrelated comorbidities [2]. The MDT typically includes a hematologist, geneticist, gastroenterologist, pediatrician, rheumatologist, and metabolic specialist. Supportive care is also provided by orthopedic surgeons, neurologists, occupational therapists, and other specialists [12, 13].

The basic premise in the management and monitoring of GD is that it is a chronic, progressive disease that ultimately leads to reduced life expectancy [1]. The goals of treatment focus on three main areas: 1) recovery of hematologic parameters, 2) reduction in visceral volumes, and 3) reduction of bone marrow infiltration with lessening of bone pain and prevention of irreversible skeletal complications as the ultimate therapeutic end-points [10, 14]. The most frequent manifestations of GD at diagnosis—according to International Collaborative Gaucher Group (ICGG) and other registries—include anemia (29%), thrombocytopenia (62%), splenomegaly (91%), bleeding (20.6%), and bone pain (57.9%) [14]. These parameters have traditionally been used as the primary outcome measures of treatment effectiveness. Treatment effect has been based on a set of therapeutic goals promulgated by the European Working Group on Gaucher Disease [15], which include elements such as pulmonary involvement, growth, and quality-of-life (QoL) [15, 16].

Current management of GD includes enzyme replacement therapy (ERT), substrate-reduction therapy (SRT), and supportive therapies. To date, approved treatments available for GD1 are the ERTs imiglucerase, velaglucerase alfa, and taliglucerase alfa, and the SRTs, eliglustat, and miglustat [12, 17]. Eliglustat is not registered in South Africa.

ERT revolutionized the treatment of GD and has markedly improved the prognoses of patients with GD1 (and GD3), allowing clinicians to establish meaningful therapeutic goals for the most common clinical manifestations of GD [8, 9]. Treatment with ERT has long been the standard of care (SOC) for GD1 [8, 18]. ERT reverses hematological and visceral manifestations of the disease and reduces the bone marrow burden of Gaucher cells resulting in the improvement of cytopenias, osteopenia, bone pain, and risk of bone crises [10]. During the first six months of ERT administration, most patients (∼90%) show a rapid improvement in the blood parameters of disease activity, the severe fatigue that accompanies GD, and QoL measures. While platelet count in patients with greatly enlarged spleens may require longer periods to respond, marked improvements continue progressively, with the majority of patients normalizing blood counts within the first 2–5 years of therapy [10, 14]. Improvement in the bone marrow and the osseous skeleton in response to ERT occur more slowly than the visceral and hematological responses; improvements in the bone mineral burden (BMB) score by 2 or more points appear to stabilize after 5 years after therapy, while near-normal BMB values and an increase in bone mineral density (BMD) could take up to 8 years [10, 12].

Although ERT significantly improves the systemic symptoms of GD, available therapies do not affect neurological manifestations of the disease or several aspects of bone disease, such as osteonecrosis, osteofibrosis, and lytic lesions, which are irreversible. However, timely initiation of ERT reduces the risk of these complications and has been shown to positively impact the growth of children. ERT has also been shown to decrease bleeding during pregnancy, delivery, and postpartum and improves overall outcomes of mothers who have suffered previous miscarriages [1, 8].

Imiglucerase and velaglucerase alfa have few side effects. Pruritis, which can be controlled by antihistamines, is the most common side effect. Antibody formation has also been reported, but in most cases patients are asymptomatic. The most common side effects reported with taliglucerase alpha were transient and included infusion reactions (headache, chest pain or discomfort, weakness, fatigue, skin redness, increased blood pressure, back pain, joint pain, and flushing), allergic reactions (angioedema, wheezing, and hypotension) and anaphylaxis. Urinary tract infection, cold-like symptoms, arthralgia, and headache have been observed in 10% of cases. Hypersensitivity reactions, including swelling under the skin, flushing, redness, rash, nausea, vomiting, and chest tightness may also occur [19].

While all current treatments for GD (ERTs and SRTs) can reverse many of the non-neurological manifestations, these therapies must be administered continually and are extremely costly, time-consuming, and invasive. Therefore, deciding when and how to begin treatment can be challenging [8].

The most recent South African guidelines for the management of GD were published in 2012 [4]. Over the past decade, new options for ERT have been approved, and miglustat became the first SRT approved in South Africa. In addition, a vast number of research and review articles on the management of GD, and at least three updated international expert consensus documents, have been published since.

The goal of this Delphi survey was to update the guidance for the management of GD1—both disease directed and adjunctive—by including novel research and advances in the treatment of GD. This paper provides consensus recommendations for the management of patients with GD1 and the term “Gaucher disease” will refer to that disorder unless otherwise indicated.

## Methods

### Composition of the steering committee and Delphi panel

Steering committee (SC) members and Delphi panelists were recruited by an independent third party (VI Research) that provided support for the entire Delphi process. Two steering committee members—one local and one international expert—with extensive experience in the treatment of GD were recruited to lead the project and assist in drafting the initial set of statements. Five local healthcare practitioners (HCPs) with experience in GD were recruited to serve on the independent panel for the completion of the Delphi survey. Each member of the panel demonstrated expertise in GD through relevant research activities, peer-reviewed publications, and/or experience in patient care. Each panelist provided independent evaluations of statements sent to them and they remained anonymous to each other throughout the process.

### Development of an initial set of statements

**Fig 1:**
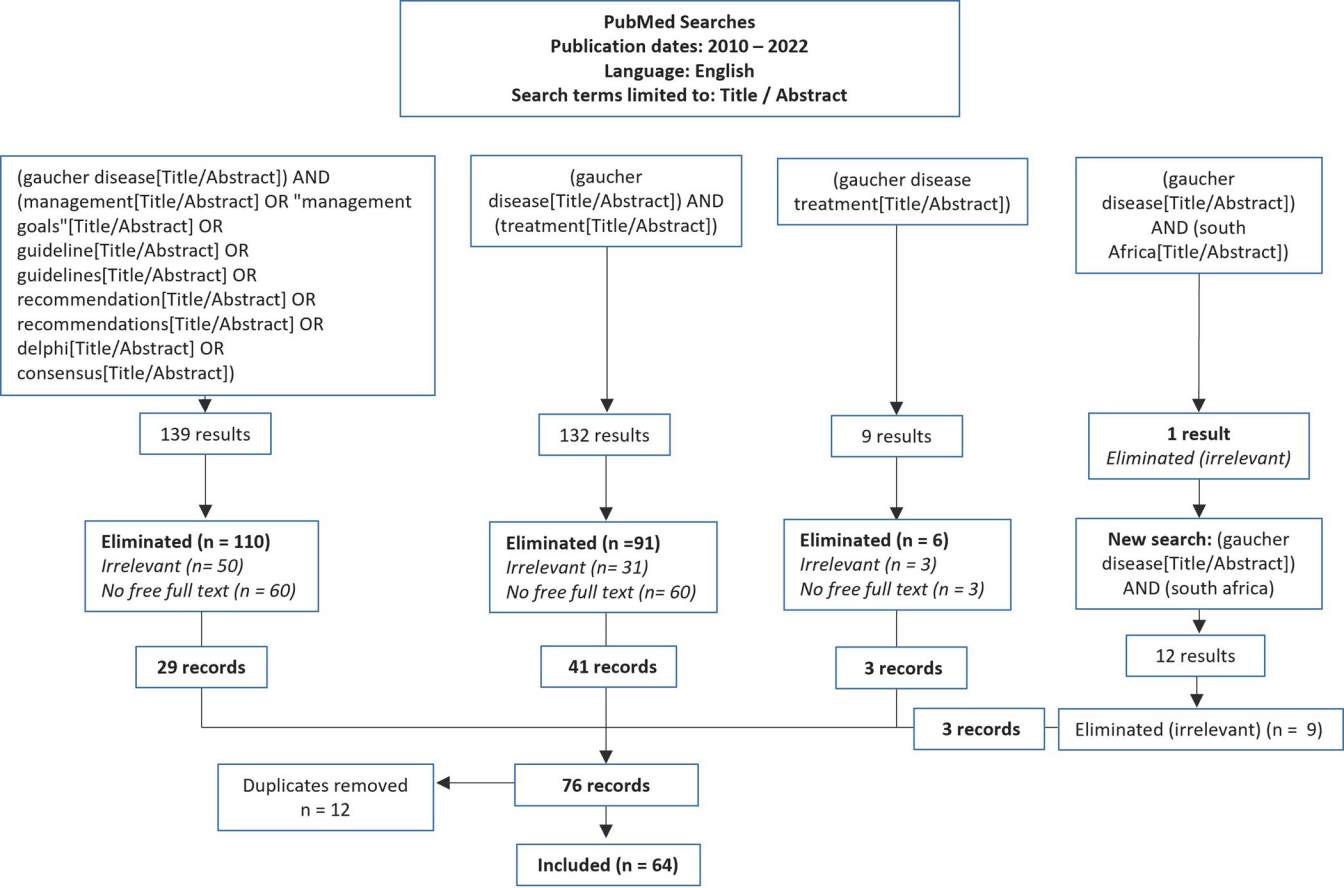
Overview of literature search.

A preliminary set of statements was drafted based on a literature search conducted by VI Research in April 2022. Four PubMed searches were performed, each covering articles published in English between 2010 to 2022 (Fig 1). Search terms were limited to the titles and abstracts and included the following combinations of terms:

1. “Gaucher disease” AND “management” OR “management goals” OR “guideline” OR “guidelines” OR “recommendation” OR “recommendations” OR “Delphi” OR “consensus”
2. “Gaucher disease AND treatment” (articles limited to reviews and systematic reviews)
3. “Gaucher disease treatment”
4. “Gaucher disease” AND “South Africa”

This search resulted in the retrieval of 283 papers. Duplicates of documents, which were found in more than one search, were manually removed from the selection. Articles were screened on title and abstract and included if they were considered relevant for the definition of management goals and/or the current standard of care (SOC) of GD1. A secondary manual search of the reference lists of the relevant articles and a comprehensive grey literature search were also carried out.

Information from selected articles guided in formulating the management goals, statements on the current SOC, and recommendations on best practice. A total of 213 statements were drafted and sent to the SC for review and revision. A final list of 205 statements and one matrix question (Tables A1-A15 in S1 Appendix) were sent to the panel for consensus development using three rounds of voting by a modified Delphi process (www.calibrum.com).

### Delphi process

All stages were overseen by an independent administrator (VI Research) who sent the statements to the panel members using a dedicated survey platform (www.calibrum.com). Panelists indicated their level of agreement on a 9-point Likert scale (1 = absolute disagreement to 9 = absolute agreement) during each round of voting. Participants were also able to provide qualitative comments in the form of free text during each round. Revisions to the statements were made by the facilitator where necessary and included in the next round of voting.

Statements were retained for the final guideline document without further voting if ≥80% of panelists indicated high agreement (7–9). All statements that scored <80% agreement and all statements that required rephrasing continued into the next round of consensus building. Statements scoring >70% low agreement (1–3) and where SC members believed it highly unlikely that the panel would reach consensus on that statement in subsequent rounds, even if the statement was amended, were removed completely. The criteria to retain a statement in the final round were ≥80% high agreement (7–9). 193 statements met this threshold after three rounds of voting and were included in the final guidance document. All votes were anonymous and weighted equally. All five panelists participated in each of the three rounds of voting. This project was not intended as a clinical guideline, case report, or to assist with the preparation of any representative statistics to be published. The project was approved by the South African Medical Association Research Ethics Committee (SAMAREC). SAMAREC is a Research Ethics Committee whose committee members provide REC services for participants involved in research studies in the Private Sector in South Africa. All panelists provided written informed consent before enrolment in the study and consented to have their names published in the Acknowledgements section of the manuscript.

## Results

After the first round of voting, consensus was reached on 186 (90.7%) statements. Nineteen individual statements and the matrix question were discussed during a virtual meeting as part one of the second round of voting. All five panelists participated in the virtual meeting. To maintain the anonymity of the Delphi panelists, the virtual meeting (via Zoom) was facilitated by VI Research, who blocked participants from turning on their cameras during the meeting. Panelists were de-identified before admission to the meeting and were told not to identify themselves at any time during the discussions. The consensus was that five of the statements should be removed, two statements rephrased, the matrix question updated, and twelve statements to remain unchanged for a revote. After the second round of voting, consensus was not reached on nine statements, all of which related to the SOC of GD in South Africa and six items in the matrix question relating to follow-up and monitoring. These were discussed during a virtual meeting (round 3). Consensus was reached on all outstanding items in the matrix question and the statements on SOC were amended to reflect the panel’s recommendations more accurately.

### Statements for which consensus was achieved

All statements for which consensus was achieved (N = 193) with their respective references are provided in Tables A1-A14 in S1 Appendix. In addition to management goals for hematological-, visceral-, skeletal- and pulmonary complications (Tables 1–4) and general well-being (Table 5), statements on the management goals in pediatric patients, pregnancy and delivery, general disease management, follow-up and monitoring, extrapyramidal disease, oral and dental manifestations, and rare complications (Tables A6-A12 in S1 Appendix) were also included in the Delphi questionnaire.

**Table 1:** Hematological complications treatment goals.

**Anemia/anemia-related symptoms**	
**Short term goals**	**Long term goals**
• Increase hemoglobin levels within 1–2 years to ≥10 g/dL (women and children) and ≥12 g/dL for men • Eliminate blood transfusion dependency • Reduce anemia-related fatigue	• Maintain improved hemoglobin values achieved after the first 1–2 years of therapy
**Bleeding tendency**	
**Short term goals**	**Long term goals**
• Increase platelet counts during the first year of treatment sufficiently to prevent surgical, obstetrical, and spontaneous bleeding • In patients with splenectomy: normalization of platelet count by 1 year of treatment • In patients with intact spleen: ○ Moderate baseline thrombocytopenia (60,000–120,000/μL): platelet count should increase by 1.5 to 2-fold by year 1 and approach low-normal levels by year 2 ○ Severe baseline thrombocytopenia (<60,000/μL): platelet count should increase by 1.5-fold by year 1 and continue to increase slightly during years 2–5 (doubling by year 2)	• Maintain platelet count of ≥100,000/mm^3^ to eliminate risk of bleeding after a maximal response has been achieved• Reduce increased bleeding tendency, whether caused by low platelet numbers, platelet defects, or coagulation abnormalities • Keep the bleeding risk of GD patients in mind in risky situations such as pregnancy and delivery, surgical interventions, and dental extractions, and establish correct preventive measures

**Table 2:** Visceral complications treatment goals.

**Spleen**	
**Short term goals**	**Long term goals**
• Avoid splenectomy • Alleviate symptoms due to splenomegaly • Eliminate hypersplenism • Reduce spleen volume to <2 to 8 times normal (or in the absence of volume measurement tools reduce spleen size by year 1–2 depending on baseline spleen volume)	• Maintain spleen volume of <2 to 8 times normal after year 1–2
**Liver**	
**Short term goals**	**Long term goals**
• Reduce liver volume to 1,0–1,5 times normal (in the absence of other hepatic disease such as viral hepatitis) by year 1–2, depending on baseline liver volume	• Maintain (near) normal liver volume after year 1–2 • Prevent liver fibrosis, cirrhosis, and portal hypertension
**Follow-up and monitoring**	
• It is generally accepted that abdominal MRI is the most reproducible approach for evaluating liver and spleen volume	

**Table 3:** Skeletal complications treatment goals.

**Mobility / skeletal involvement**	
**Short term goals**	**Long term goals**
• Lessen bone pain that is not related to irreversible bone disease within 1–2 years • Decrease bone marrow (BM) involvement, as measured by a locally used scoring system (bone marrow burden (BMB) score or Düsseldorf Gaucher score (DGS) in patients without severe irreversible bone disease) at baseline • Increase bone mineral density (BMD) by 2 years in adult patients with a T-score below -2.5 at baseline	• Prevent bone complications (avascular necrosis, bone crises, bone infarcts, and pathological fractures) • Prevent osteopenia and osteoporosis (i.e., maintain BMD T-scores (DEXA) of >-1) • Prevent osteonecrosis and subchondral joint collapse • Prevent chronic use of analgesic medication for bone pain • Maintain normal mobility or, if impaired at diagnosis, improve mobility • Increase physical activity • Improve trabecular BMD commensurate with patient’s age (z-score, not t-score) by 3 to 5 years
**Therapy (bone involvement)**	
• Therapy should address GD-specific and conventional challenges to bone, and include generally appropriate attention to dietary (calcium and vitamin D) and lifestyle factors • Patients with bone mineral deficit may benefit from anti-osteoporotic drugs, particularly bisphosphonates, in addition to specific therapy (ERT) for GD.	

**Table 4:** Pulmonary complications treatment goals.

**Pulmonary complications**
• Prevent or improve pulmonary disease, such as pulmonary hypertension (PH) and hepatopulmonary syndrome (HPS) • Reverse hepatopulmonary syndrome and dependency on oxygen • Improve functional status and QOL • Prevent sudden death • Prevent pulmonary disease by timely initiation of ERT and avoidance of splenectomy
**Lung infiltration**
• Consider using a bronchodilator during winter months; monitor patients using PFTs • Patients with decreased forced vital capacity (FVC), DLCO <80%, severe chest infections, and/or recurrent hospital admissions due to chest infections should be referred to a pulmonologist to guide further management • Antibiotics, when indicated, should be started promptly • Influenza, pneumococcal, and SARS-CoV2 vaccination are recommended for patients with symptomatic lung disease • Oxygen therapy is indicated for patients with GD and lung infiltration causing severe hypoxia • ERT in people with compromised lung expansion or hepatopulmonary syndrome (HPS)
**Pulmonary hypertension**
• Ameliorate PH with the use of ERT and adjunctive medications as needed • Management should be guided by specialist respiratory and cardiology teams • Administer vasodilators, with careful monitoring for drug-induced pulmonary edema

**Table 5:** General well-being treatment goals.

**General well-being**	
**Short term goals**	**Long term goals**
• Improve scores from the baseline of a validated QOL instrument within 2–3 years or less, depending on the disease burden • Improve or restore physical function for carrying out normal daily activities and fulfilling functional roles	• Maintain good QOL measured by validated instruments that are easily applicable in the clinic and are of value in making treatment decisions. • Maintain normal participation in school and work activities • Normalize life expectancy • Provide psychological care to reduce the mental and emotional impact of GD and life-long treatment on patients and their families
**Fatigue**	
• Reduce fatigue (not anemia related) as measured by a validated fatigue scoring system • Fatigue can be an important factor when evaluating and treating patients with GD and this domain should be measured before treatment and incorporated as an important treatment goal. • Fatigue assessment instruments must be easy for individuals to understand and complete, must be capable of measuring the impact of therapeutic interventions, and have robust psychometric properties. • The tool should be disease-specific, age-appropriate, culturally appropriate, and sensitive for detecting changes that are clinically meaningful for patients	

#### Hematological-, visceral,—skeletal-, and pulmonary involvement

The primary treatment goals relating to anemia, bleeding tendency, liver- and spleen volume, and bone- and lung involvement focus on achieving and maintaining (near) normal values and preventing complications. Therapy should address GD-specific challenges, including eliminating the need for blood transfusions, splenectomy, and chronic use of analgesics, and reducing debilitating symptoms like fatigue and bone pain.

#### General well-being

Therapy should improve patients’ quality of life and physical and mental well-being to allow them to carry out normal daily activities and maintain normal participation in school and work activities. Management should also include providing psychological care to reduce the mental and emotional impact of GD and lifelong treatment on patients and their families.

#### Pediatric patients

In children, treatment goals include normalizing growth, achieving normal onset of puberty, and attaining normal or ideal peak skeletal mass. Cortical and bone mineral density should be increased by year two after initiation of treatment. In symptomatic children, treatment should begin early to avoid irreversible visceral or skeletal damage and/or long-term growth and development issues. Asymptomatic children should be monitored 6-monthly to assess the rate of progression. When a child in a family is diagnosed with GD, siblings should be screened.

#### Pregnancy and delivery

Management goals for pregnant patients include preventing GD-related complications during pregnancy and delivery and monitoring hemostatic function and cellular counts to minimize bleeding risk. Continuation of ERT at the pre-pregnancy dose is not associated with any harm to the fetus or mother and may be continued, as it may decrease the risk for postpartum hemorrhage and the requirement for red blood cell transfusions. SRT is not recommended during pregnancy as there is a paucity of data on fetal outcomes. Asymptomatic patients should not begin therapy unless necessary. Genetic counseling is an important component of supportive care for families affected by a diagnosis of GD. Carrier testing, as well as pre-and post-test counseling, must be done to ensure that the family understands the medical facts, associated risks, and the options available to them.

#### Parkinson’s disease, oral manifestations, and rare complications

There are several less commonly reported disease manifestations of GD which are often under-recognized. These include Parkinson’s disease, oral and dental manifestations, Gaucheroma, and lymphadenopathy. Patients with or at risk of developing these conditions must be identified early to optimize treatment and management regimens.

*Parkinson’s disease (PD)*. The mutations associated with GD1 might predispose carriers and patients with GD to Parkinson’s disease [3]. The HCP should counsel GD patients at diagnosis, or as soon as possible during follow-up, about the increased risk of PD; however, patients should be advised that not all GD patients develop PD (<5%). From the age of 35–40 years, HCPs should monitor patients for non-motor and motor symptoms or signs suggestive of PD in 12-monthly intervals and refer the patient to a neurologist when presenting with at least one clear motor sign alone or in the presence of any non-motor symptoms or signs with impact on the QOL.

*Dental and oral manifestations*. Although dental involvement is a less common manifestation of GD, it is nonetheless imperative for dental practitioners to be aware of this disease, and to be familiar with the possible oral and dental complications that could develop.

*Gaucheroma and GD lymphadenopathy*. Manifestations like Gaucheroma and GD lymphadenopathy can be progressive and life-threatening and can affect people of all ages with a range of GD phenotypes who can be slow or fail to respond to current treatments. GD patients should be assessed for Gaucheroma at baseline and HCPs should ensure regular imaging is performed. MRI/CT imaging is preferable, if available, to monitor Gaucheroma and GD lymphadenopathy. If ultrasound is used, the radiologist should be experienced to reduce user errors. Biopsy should be avoided in GD lymphadenopathy and surgical removal is not indicated. The dosing frequency of ERT may need adjusting in severely affected patients.

#### General disease management

General disease management of GD includes:

- Early detection of hematological malignancies, solid tumors, parkinsonism/Parkinson’s disease/Parkinsonian tremor, peripheral neuropathy, insulin resistance and type 2 diabetes mellitus and signs and symptoms indicative of GD3, such as eye movement abnormalities
- Monitoring treated patients with Lyso-Gb1/Lyso-GL1 in addition to the usual standard of care for predicting long-term clinical outcomes in patients with GD
- Using chitotriosidase as a useful biomarker for serial monitoring of individual patients receiving ERT in the context of other clinical indicators of disease activity
- In the follow-up of patients, peripheral blood cell counts are mandatory parameters for periodic assessment, whether the patients are on treatment or not
- In situations of anemia not resolved by treatment, the morphology of red blood cells must be monitored to rule out megaloblastosis; iron studies, vitamin B12, and folate levels in the blood must be determined periodically
- In cases with persistent thrombocytopenia, associated immune thrombocytopenia must be ruled out. It should become mandatory to test for GD in any patient being considered for splenectomy when the cause of splenomegaly has not been established.

The panel agreed that managing patients with GD requires an MDT approach that includes disease-specific treatments, supportive care, and proper education of the patient and his family about the disease and therapy.

#### Follow-up and monitoring

All patients with GD, irrespective of the disease-specific treatment, must be followed up for evaluation of the disease status and assessment of all systems likely to be affected in GD1. This should include comprehensive and reproducible evaluation and monitoring of all clinically relevant aspects, including complete physical examination, measurements of hemoglobin concentration, platelet count, and disease-related biomarkers, as well as radiologic imaging to assess liver and spleen volumes, and skeletal involvement. Evaluation of patient-reported QoL is also recommended using the 36-item Short Form Health Survey (SF-36) every 12 months.

Patients should be followed up based on the clinical features at the time of presentation with a detailed evaluation at each visit to assess the improvement of disease manifestations as a response to therapy. Recommendations on the frequency of specific tests for monitoring patients with GD1 are listed in Table A9.2 in S1 Appendix. It is important that physicians set realistic expectations for improvement of the disease and discuss this with the patient and their family before initiating treatment.

#### Choice of therapy

*First line treatment*. ERT is proven to be the standard of care in the therapeutic management of symptomatic patients with GD1. There are currently three ERTs available in South Africa: imiglucerase, velaglucerase alfa, and taliglucerase alfa. It is up to the treating physician to decide which ERT to use as there is no demonstration of superiority between the three available ERTs.

*Second line treatment*. Substrate reduction therapy is indicated for patients who are not eligible for ERT. Currently, only miglustat is registered in South Africa. Miglustat is indicated for the oral treatment of adult patients with mild to moderate GD1, but only in those patients for whom ERT is unsuitable. It is not approved for use in children or patients with severe disease. Miglustat is also associated with more frequent adverse events than ERTs, most notably diarrhea, weight loss, tremor, and peripheral neuropathy. Eliglustat, a newer SRT (not registered in South Africa yet) has an improved risk/benefit profile compared to miglustat and might be a viable alternative to ERT for eligible patients once it becomes available.

*Dosing and administration*. Recommendations on dosing and administration of ERT and SRT are shown in Table 6.

**Table 6:** Treatment options available for treating patients with GD1 in South Africa.

	Drug class	Available drugs	Recommended dose	Comments*
**First line treatment**	ERT	Imiglucerase	• Adult dose: 15 u/kg EOW • Children: 30 u/kg EOW • Patients in whom immediate disease control is a priority: consult with specialists and subject experts to titrate patient’s therapy according to clinical indicators	• It is up to the treating physician to decide which ERT to use as there is no demonstration of superiority between the three available ERTs • The optimal dose of ERT needs to be individualized and will depend upon body weight and overall response to treatment. The weight of the patient is checked at each assessment and the dose is adjusted according to the bodyweight
		Velaglucerase alfa		
		Taliglucerase alfa		
**Second line treatment**	SRT	Miglustat	• Adults dose: 100 mg capsule TDS • It may be necessary to reduce the dose to one 100 mg OD or BD in some patients due to adverse reactions	• Indicated for the oral treatment of adult patients with mild to moderate GD1, but only in those patients for whom ERT is unsuitable. • Not approved for use in children or patients with severe disease

* Therapy should be directed by physicians who are knowledgeable in the management of Gaucher disease.

## Discussion

The management goals of GD have not changed significantly over the past two decades. In general, the management goals presented in this paper are in line with existing literature on the subject, specifically the therapeutic goals as proposed by the European Working Group on Gaucher Disease and others [16, 20]. Some statements included in existing literature required amendment before consensus was reached. For example, “Mucolytics, guided by a respiratory team, are recommended for patients with decreased FVC, Dlco <80%, severe chest infections and/or recurrent hospital admissions due to chest infections” was changed to “Patients with decreased FVC, DLCO <80%, severe chest infections and/or recurrent hospital admissions due to chest infections should be referred to a pulmonologist to guide further management” as the panel agreed that by the time there is significant lung involvement, a patient should be under a pulmonologist’s care. “Monitoring treated patients with Lyso-Gb1 in addition to the usual standard of care for predicting long-term clinical outcomes in patients with GD” was changed to include “Lyso-Gb1/Lyso-GL1” to limit confusion. Though the two terms are synonymous, most laboratories in South Africa use the term Lyso-GL1 in reference to this test, whereas Lyso-Gb1 seems to be the preferred term in other published literature.

Statements relating to the current standard of care and proposed standard of care for GD1 in South Africa were discussed in depth during the consensus procedure and the panel agreed that the original statements included in the survey (refer to Tables A15 and A16 in S1 Appendix) should either be amended or removed and replaced with recommendations that more accurately reflect the outcome of those discussions. For example, the expert panel agreed that there is insufficient evidence to support the widespread administration of ERT every four weeks. They recommend that ERT be administered every other week (EOW) for adults and children with GD1, and the consensus was that statements recommending 4-weekly dosing be removed.

The panel further agreed that there is an argument to be made for low-dose ERT initiation. Several other countries are advocating for this, with dose increases where clinically indicated. In a resource-constrained country like South Africa, the expert panel recommends a standard dose of 15 units/kg EOW for adult patients and 30 units/kg EOW for pediatric patients. Statements recommending high-dose treatment were removed or rephrased, and to prevent treating physicians from reverting to the higher dose, range values for dosing were removed. The consensus recommendation for patients in whom immediate disease control is a priority (i.e., moderate to severe disease including those with life-threatening complications such as hepatopulmonary syndrome and pulmonary hypertension) is that the treating physician should consult with specialists and subject experts to titrate the patient’s therapy according to clinical indicators.

Other statements that were removed relate to the indication of lung transplantation in patients with end-stage lung disease secondary to GD or end-stage GD1-related pulmonary hypertension, mainly because lung transplantation is an extreme intervention that would be reserved for only the most severe cases and only considered after extensive consultation by a specialist team, including disease area experts, pulmonologists and surgeons where the individual patient’s entire clinical picture would be considered. A statement on the use of heel ultrasound to assess bone involvement was removed as some panelists indicated that they were unfamiliar with this technique and could therefore not make a recommendation on its indication. After deliberation, the panel agreed that heel ultrasound is not a common investigation and would be very operator dependent, i.e., there may only be a few people who would be comfortable doing this investigation to produce accurate results, and that it would be inappropriate to make a statement about such a niche investigation to apply across the board.

The statement regarding a patient’s right to refuse to be informed on the risk of Parkinson’s Disease was also removed, as panel members argued that not only is it difficult to implement, but also that the statement is contradictory; the patient won’t know what not to be informed about, therefore, the patient will have to be told that there is a potential risk before they can refuse to be informed. Conversely, if the patient proactively refuses to be informed about the risk of PD, it means that he/she is already aware of the risk. The statement regarding cessation of treatment "if the health and well-being of medical and/or nursing staff are placed under significant threat as a result of the actions or lifestyle of the patient” was also removed as the panel failed to see the relevance of the statement.

Additional management goals and general statements/recommendations on sound clinical practice in the management of GD, obtained from new research publish since 2010, and the panelists’ own clinical experience has been added to develop a comprehensive consensus document on the goals of treatment of GD1, for example, panel consensus was that although it is generally accepted that abdominal MRI is the most reproducible approach for evaluating liver and spleen volume, ultrasound is also acceptable for follow-up of spleen and liver size when MRI is not available/affordable.

After three rounds of voting, the expert panel reached consensus on a comprehensive set of management goals by using the Delphi technique. The Delphi method is a widely-used tool in consensus procedures that facilitates the exchange of information and ideas while promoting careful and in-depth thinking [21]. Panel members contribute a wide range of knowledge and experience to the decision-making processes, where expert participants are more likely to provide reasoned, independent, and well-considered opinions in the absence of exposure to persuasively stated opinions of others, as is sometimes seen, for example, in focus groups [21]. Participants are further provided the opportunity to retract, alter or add to their original answers with the benefit of considered thought. One of the main advantages of the Delphi method is anonymity, as this allows participants to express opinions they might not otherwise be able to express [21].

However, anonymity may also be a limitation of the Delphi method, as the promise of anonymity may lead to a lack of accountability of opinions expressed and lead to rash decisions [21]. While the Delphi method provides consensus of expert opinion without bias, the consensus approach may lead to unimaginative versions of the best opinion. In addition, using a group of experts who are purposefully rather than randomly selected, relies on subjective opinions of individuals (although informed by objective information) which may not always be representative of the target population [21].

Patient access and reimbursement of ERT and/or SRT for the treatment of GD1 was beyond the scope of this manuscript, not least because the subject of access to and funding of these drugs for GD1 (and other rare diseases, in general) is a controversial and often contentious issue within the South African payer landscape. Unpacking the complexities of this topic would require consultation with various stakeholders, including government, governing bodies, legal experts, patients, and patient advocacy groups, which could warrant an entire study in itself.

The primary objective of this manuscript was to get expert consensus on the management goals of GD1 in South Africa. However, the results obtained from this Delphi survey, including the expert recommendations from the panel, should always be taken as a guideline. The wide clinical variability of GD requires the application of personalized medicine, considering all variables and different disease scenarios in order to adjust to the individual patient’s needs, the patient’s perception of their symptoms, and how the disease affects them daily. The hope is that this consensus document can be used to aid decision-makers as part of a rational, evidence-based decision-making process to inform future policy decisions on funding clinically appropriate treatments for patients with GD1.

## Conclusion

The rarity of GD, its clinical heterogeneity, unpredictable progression rates, and variable nature of symptoms may still represent a challenge for many clinicians who are not familiar with how to accurately diagnose, treat or monitor these patients. Untreated or sub-optimally treated GD1 patients may experience permanent disability and reduced life expectancy.

**Fig 2:**
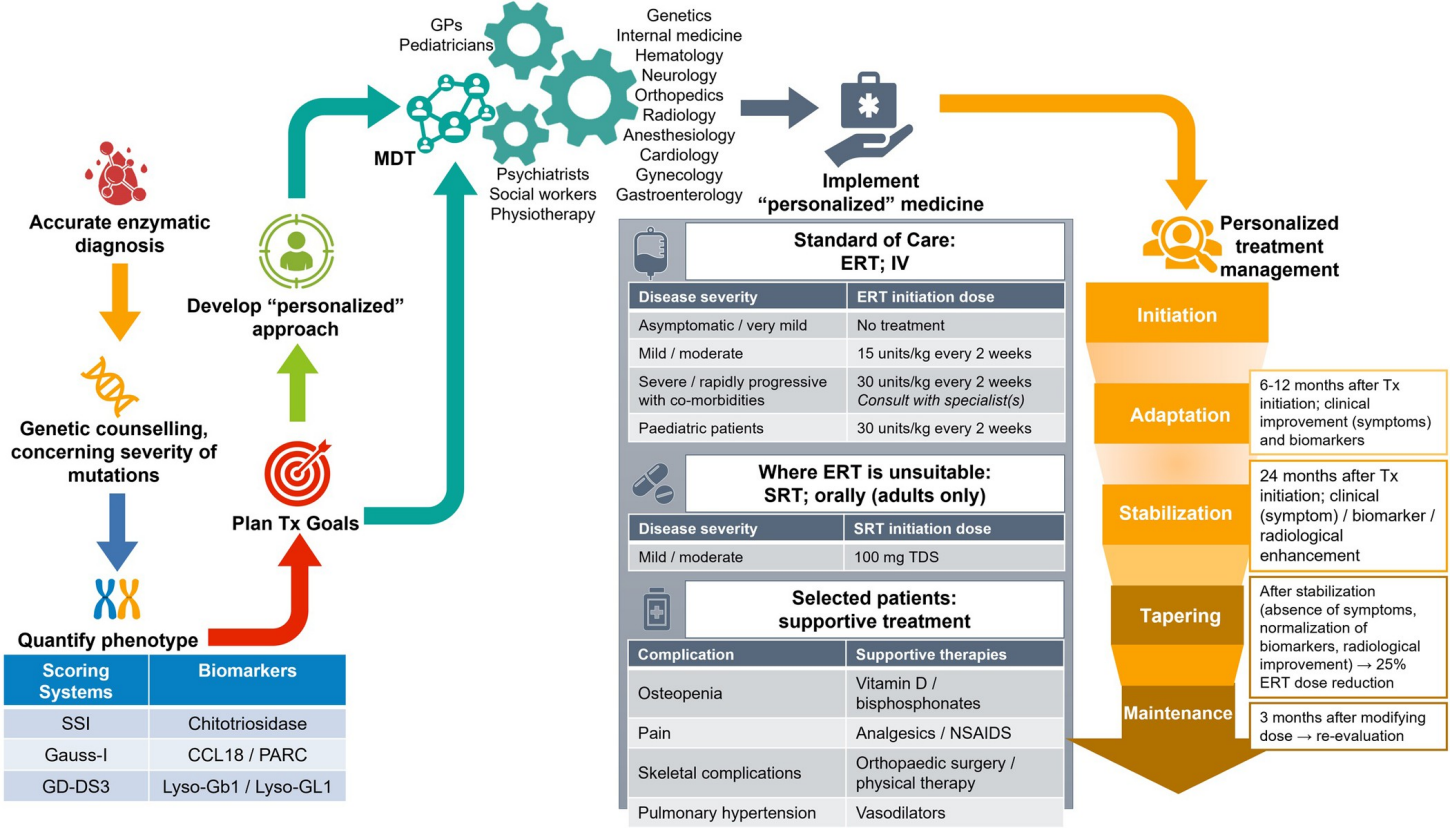
Management of GD1. (Adapted from Torralba-Cabeza *et al*. [22]) CCL18: chemokine ligand 18; ERT: enzyme replacement therapy; GauSS-I: Gaucher Severity Score Index for type I patients; GD-DS3: Gaucher Disease Type I severity scoring system; IV: intravenous; Lyso-Gb1/Lyso-GL1: glucosylsphingosine; MDT: multidisciplinary team; NSAIDS: non-steroidal anti-inflammatory drugs; PARC: pulmonary and activation-regulated chemokine; SRT: substrate reduction therapy; SSI: Severity Score Index; TDS: three times daily; Tx: treatment.

This expert consensus document summarizes current published information related to the treatment goals of GD1. The Delphi exercise resulted in high-level guidance concerning management goals, and the use of current therapies and adjunctive interventions in GD1 to assist HCPs in their decisions about appropriate management, as we strive for earlier diagnosis and optimized treatment options (Fig 2). The management goals and best practice statements included herein might be used to inform an update to future South African guidelines on the disease.

## Supporting information

S1 Appendix Complete list of statements included in the Delphi questionnaire. (PDF)
