# Supplementary material for: Management goals of type 1 Gaucher disease in South Africa: An expert Delphi consensus document on good clinical practice
Source: PLoS One. 2023 Aug 22;18(8):e0290401. doi: 10.1371/journal.pone.0290401 (PMC10443848; doi:10.1371/journal.pone.0290401)
Supplement: S1 Appendix — (PDF) [file pone.0290401.s001.pdf]

S1 Appendix.

Management goals of type 1 Gaucher disease in South Africa: an expert Delphi consensus document on good clinical practice.

Complete list of statements included in Delphi questionnaire.

Abbreviations

**BMB**	bone marrow burden
**BMD**	bone mineral density
**CT**	computed tomography
**CXR**	chest X-ray
**DEXA**	dual-energy X-ray absorptiometry
**DGS**	Düsseldorf Gaucher Score
**DLCO**	diffusing capacity of the lungs for carbon monoxide
**ECG**	electrocardiogram
**EOW**	every other week
**ERT**	enzyme replacement therapy
**FVC**	forced vital capacity
**GD**	Gaucher disease
**HPS**	hepatopulmonary syndrome
**MRI**	magnetic resonance imaging
**PD**	Parkinson's disease
**PH**	pulmonary hypertension
**PLE**	protein-losing enteropathy
**QOL**	quality of life
**SRT**	substrate reduction therapy

A.Management goals for which consensus was achieved.

The consensus management goals (N = 193) are presented in Tables A1-A14.

Table A1. Hematological complications treatment goals.

Category	Time frame	Goal	Reference(s)
**Anemia/anemia-related symptoms**	**Short term**	Increase hemoglobin levels within 1-2 years to ≥10 g/dL (women and children) and ≥12 g/dL for men	[1-6]
		Eliminate blood transfusion dependency	[1-5]
		Reduce anemia-related fatigue	[4]
	**Long term**	Maintain improved hemoglobin values achieved after the first 1-2 years of therapy	[1-5]
**Bleeding tendency**	**Short term**	Increase platelet counts during the first year of treatment sufficiently to prevent surgical, obstetrical, and spontaneous bleeding	[1-5]
		In patients with splenectomy: normalization of platelet count by 1 year of treatment	[1-3, 5]
		In patients with intact spleen:<br>Moderate baseline thrombocytopenia (60,000 - 120,000/µL): platelet count should increase by 1.5 to 2-fold by year 1 and approach low-normal levels by year 2;<br>Severe baseline thrombocytopenia (<60,000/µL): platelet count should increase by 1.5-fold by year 1 and continue to increase slightly during years 2-5 (doubling by year 2)	[2, 5, 6]
	**Long term**	Maintain platelet count of ≥100,000/mm^3 to eliminate risk of bleeding after a maximal response has been achieved	[1, 4, 5]
		Reduce increased bleeding tendency, whether caused by low platelet numbers, platelet defects or coagulation abnormalities	[2, 3]
		Keep the bleeding risk of GD patients in mind in risky situations such as pregnancy and delivery, surgical interventions, and dental extractions, and establish correct preventive measures	[7]

Table A2. Visceral complications treatment goals.

Category	Time frame	Goal	Reference(s)
Spleen	Short term	Avoid splenectomy (may be necessary during life-threatening hemorrhagic events)	[1-6]
		Alleviate symptoms due to splenomegaly (abdominal distension, early satiety, new splenic infarction detected by ultrasound, hepatic stretching of ligaments)	[1-6]
		Eliminate hypersplenism	[1-3, 5]
		Reduce spleen volume to <2 to 8 times normal (or in the absence of volume measurement tools reduce spleen size by year 1-2 depending on baseline spleen volume)	[1, 3, 5, 6]
	Long term	Maintain spleen volume of <2 to 8 times normal after year 1-2	[1-5]
Liver	Short term	Reduce liver volume to 1,0-1,5 times normal (in the absence of other hepatic disease such as viral hepatitis) by year 1-2, depending on baseline liver volume	[1-5]
	Long term	Maintain (near) normal liver volume after year 1-2	[1-3]
		Prevent liver fibrosis, cirrhosis, and portal hypertension	[3, 4]
Follow-up and monitoring		It is generally accepted that abdominal MRI is the most reproducible approach for evaluating liver and spleen volume	[4]
		Ultrasound is also acceptable for follow-up of spleen and liver size when MRI is not available/affordable	Expert consensus

Table A3. Skeletal complications treatment goals.

Category	Time frame	Goal	Reference(s)
**Mobility / Skeletal involvement**	**Short term**	Lessen bone pain that is not related to irreversible bone disease within 1-2 years	[1, 3, 5, 6]
		Decrease bone marrow involvement, as measured by a locally used scoring system (bone marrow burden (BMB) score or Düsseldorf Gaucher score (DGS) in patients without severe irreversible bone disease) at baseline	[3]
		Increase bone mineral density (BMD) by 2 years in adult patients with a T-score below -2.5 at baseline	[1, 3]
	**Long term**	Prevent bone complications (avascular necrosis, bone crises, bone infarcts and pathological fractures)	[1, 3, 5, 6]
		Prevent osteopenia and osteoporosis (i.e., maintain BMD T-scores (DEXA) of >-1)	[3]
		Prevent osteonecrosis and subchondral joint collapse	[4, 5]
		Prevent chronic use of analgesic medication for bone pain	[3]
		Maintain normal mobility or, if impaired at diagnosis, improve mobility	[3, 4]
		Increase physical activity	[3]
		Improve trabecular BMD commensurate with patient’s age (z-score, not t-score) by 3 to 5 years	[5, 6]
	**Follow-up and monitoring**	The severity of skeletal involvement in GD1 is variable, but associated with significant pain, disability, and reduced quality of life (QOL); thus, all patients should be monitored regularly for the onset and progression of skeletal pathology	[8]
		MRI is the method of choice for evaluating bone marrow infiltration	[8]
		MRI is valuable for assessment of structural bone abnormalities	[2]
		DEXA is the method of choice for measuring BMD	(2)
		For pediatric patients, DEXA must be performed at a center that uses pediatric reference values	[2]
		DEXA measurements should be done at bone sites that are free of osteonecrosis	[2]
		DEXA is the method of choice for evaluating osteopenia/osteoporosis	[2]
	**Therapy (bone involvement)**	Therapy should address GD-specific and conventional challenges to bone, and include generally appropriate attention to dietary (calcium and vitamin D) and lifestyle factors	[9]
		Patients with bone mineral deficit may benefit from anti-osteoporotic drugs, particularly bisphosphonates, in addition to specific therapy (enzyme replacement therapy (ERT)) for GD	[8]
		Bone marrow disease is rapidly responsive to GD-specific therapy	[9]

Table A4. Lung involvement and pulmonary complications treatment goals.

Category	Goal	Reference(s)
**Pulmonary complications**	Prevent or improve pulmonary disease, such as pulmonary hypertension (PH) and hepatopulmonary syndrome (HPS)	[1, 3]
	Reverse hepatopulmonary syndrome and dependency on oxygen	[4, 5]
	Improve functional status and QOL	[5]
	Prevent sudden death	
	Prevent pulmonary disease by timely initiation of ERT and avoidance of splenectomy	
**Lung Infiltration**	Consider using a bronchodilator during winter months; monitor patients using pulmonary function tests (PFT)	[10]
	Patients with decreased forced vital capacity (FVC), DLCO <80%, severe chest infections and/or recurrent hospital admissions due to chest infections should be referred to a pulmonologist to guide further management	
	Antibiotics, when indicated, should be started promptly	
	Influenza, pneumococcal and SARS-CoV2 vaccination are recommended for patients with symptomatic lung disease	
	Oxygen therapy is indicated for patients with GD and lung infiltration causing severe hypoxia	
	ERT in people with compromised lung expansion or HPS	
**Pulmonary hypertension**	Ameliorate PH (with use of ERT + adjunctive medications as needed)	[4, 5]
	Management should be guided by specialist respiratory and cardiology teams	
	Administer vasodilators, with careful monitoring for drug-induced pulmonary edema	[10]

Table A5. General well-being and quality of life treatment goals.

Category	Time frame	Goal	Reference(s)
**General well-being**	**Short term**	Improve scores from baseline of a validated QOL instrument within 2-3 years or less, depending on disease burden	[1-3, 6]
		Improve or restore physical function for carrying out normal daily activities and fulfilling functional roles	[1-3]
	**Long term**	Maintain good QOL measured by validated instruments that are easily applicable in the clinic and are of value in making treatment decisions.	[3, 11]
		Maintain normal participation in school and work activities	[3]
		Normalize life expectancy	[3]
		Provide psychological care to reduce mental and emotional impact of GD and life-long treatment on patients and their families	[3, 4]
**Fatigue**		Reduce fatigue (not anemia related) as measured by a validated fatigue scoring system	[2, 3]
		Fatigue can be an important factor when evaluating and treating patients with GD and this domain should be measured before treatment and incorporated as an important treatment goal.	[12]
		Fatigue assessment instruments must be easy for individuals to understand and complete, must be capable of measuring the impact of therapeutic interventions, and have robust psychometric properties.	[12]
		The tool should be disease specific, age appropriate, culturally appropriate, and sensitive for detecting changes that are clinically meaningful for patients	

Table A6. Management goals for pediatric patients with GD1.

Category	Goal	Reference(s)
Pediatric patients	Normalize growth such that the height of the patient is in line with target height, based upon population standards and parental height, within 2 years of treatment	[1, 2, 6]
	Achieve normal onset of puberty	[1-3, 5, 6]
	Attain normal or ideal peak skeletal mass	[1, 3, 5]
	Increase cortical and bone mineral density by two years	[5]
	Healthy levels of exercise and physical activity should also be encouraged in children with GD where possible	[13]
	It is recommended that treatment begin early in symptomatic children with GD1 and GD3 to avoid irreversible bony and visceral damage as well as other long-term growth and development issues	[14]
	When a child in a family is diagnosed with GD, siblings should be screened. There is wide phenotypic variation even among siblings; hence, pre-symptomatic diagnosis is not in itself an indication for therapy	[11, 14]
	Asymptomatic children should be monitored at 6-monthly intervals to assess rate of progression and development of cytopenia, splenomegaly and skeletal disease, so treatment can be initiated quickly should signs and symptoms develop	[11, 14]

Table A7. Management goals for GD1 during pregnancy and delivery.

Category	Goal	Reference(s)
**Pregnancy and delivery**	Prevent GD related complications during pregnancy and delivery	[3, 4]
	Hematological abnormalities associated with GD are of special importance during pregnancy, requiring the monitoring of hemostatic function and cellular counts to minimize the bleeding risk	[7]
	Asymptomatic patients should not begin therapy unless necessary	[14]
**Genetic counselling and prenatal diagnosis**	Genetic counselling is an important component of supportive care for any family and best provided by a healthcare professional well versed in these aspects of care	[2]
	Affected persons must also understand the risk of transmission of a mutated gene to their offspring and carrier testing is therefore important for these families	[2]
	Pre-test and post-test counselling must be done to ensure that the family understands the medical facts, the risk of disease in each pregnancy, the reproductive options to prevent recurrence, implications of giving birth to a child with GD, and the options available to them if the fetus is identified to be affected	[6]
	Parents of affected individuals, individuals themselves when they reach an age of understanding, siblings of carriers or affected individuals, and spouses / potential spouses should be included	[2]
	Whereas enzyme activity is a reliable method for the diagnosis of GD, its use for identifying carriers is limited. The best method for carrier testing and prenatal diagnosis is mutation analysis	[2]
	Prenatal testing is best performed by initial genotype determination in the affected proband, confirmation of obligate carrier state in the parents or by testing of the family member under investigation	[6]

Table A8. General disease management of GD1.

Category	Goal	Reference(s)
**General disease management**	Early detection of hematological malignancies, including multiple myeloma, lymphoma, and amyloidosis	[3]
	Early detection of solid tumors, including hepatocellular carcinoma and renal cell carcinoma	[3]
	Early detection of parkinsonism / Parkinson's disease	[3]
	Early detection of insulin resistance and type 2 diabetes mellitus	[3]
	Proper education of the patient and his family about the disease and therapy	[3]
	Early detection of signs and symptoms indicative of GD3, such as eye movement abnormalities	[3]
	In non-neuronopathic disease, physical examination should also include a search for symptoms and signs of Parkinsonian tremor and peripheral neuropathy	[15]
	Managing patients with GD requires a multidisciplinary team approach that includes disease specific treatments as well as supportive care	[4]
	In GD patients undergoing surgery, it is important to carry out a hemostatic evaluation , to compensate for deficiencies	[7]
	Monitoring treated patients with Lyso-Gb1/Lyso-GL1 in addition to the usual standard of care for predicting long-term clinical outcomes in patients with GD	[4]
	Chitotriosidase is a useful biomarker for serial monitoring of individual patients receiving ERT; but it should be used in the context of other clinical indicators of disease activity	[6]
	In the follow-up of patients, peripheral blood cell counts are mandatory parameters for periodic assessment, whether the patients are on treatment or not	[7]
	In situations of anemia not resolved by treatment, the morphology of red blood cells must be monitored to rule out megaloblastosis, and vitamin B12 and folate levels in the blood must be determined periodically	
	In cases with persistent thrombocytopenia, an associated immune thrombocytopenia must be ruled out	
	It should become mandatory to test for GD in any patient being considered for splenectomy when the cause of splenomegaly has not been established	[11]

Table A9.1. Follow-up and monitoring of patients with GD1.

Category	Goal	Reference(s)
**Follow-up and monitoring**	It is essential to set realistic expectations on improvement of the disease phenotype and discuss this with the family prior to initiation of therapy	[6]
	Patients are followed up based on the clinical features at the time of presentation with a detailed evaluation at each visit to assess the improvement of disease manifestations as a response to therapy	
	All patients with GD, irrespective of the disease-specific treatment, must be followed up for evaluation of the disease status; this should include assessment of all systems likely to be affected in GD	
**Follow-up imaging**	**Plain radiology**:<br>• skeletal survey when clinically indicated for acute bone crisis or diagnosis of fracture<br>• chest X-ray (CXR) for suspected pulmonary involvement	[2]
	• **Ultrasound:** organ measurement (liver and spleen size when volumetric MRI not available), gall stones, portal hypertension or chronic liver disease, renal involvement<br>• **MRI:** see Table A9.2<br>• **DEXA:** see Table A9.2	
**Cardiopulmonary evaluation**	CXR, PFT and electrocardiogram (ECG) at baseline and as indicated; echocardiography in selected patients to confirm or refute the suspicion of PH or cardiomyopathy	[2]

Table A9.2. Minimum recommendations for monitoring patients with GD1.

	Patients not on ERT			Patients on ERT		Patients on ERT			Patients on ERT
				Therapeutic goals not achieved		Achieved therapeutic goals			At time of dose change or significant clinical complications
**Frequency (every X months)**	**12**	**12-24**	**24-36**	**3**	**12**	**12-24**	**24-36**	**36**	
Comprehensive physical examination	**X**			**X**		**X**			**X**
SF-36 (QOL) survey	**X**				**X**	**X**			**X**
**Blood tests**									
Haemoglobin	**X**			**X**		**X**			**X**
Platelet count	**X**			**X**		**X**			**X**
Biochemical markers	**X**			**X**		**X**			**X**
**Visceral**									
Spleen volume (ultrasound where volumetric MRI or CT scan is not available)		**X**			**X**	**X**			
Liver volume (ultrasound where volumetric MRI or CT scan is not available)		**X**			**X**	**X**			
**Skeletal**									
MRI of entire femora (coronal; T1 and T2 weighted)		**X**			**X**		**X**		
DEXA (lumbar spine and femoral neck)			**X**		**X**			**X**	

Table A10. Management goals for extrapyramidal disease or risk of Parkinson's Disease (PD) in patients with GD1.

Category	Goal	Reference(s)
**Communication of PD risk in GD patients**	The issue of the risk for PD among GD patients is still an area of active investigation and healthcare providers should keep up to date on this issue	[15]
	The healthcare provider should counsel GD patients at diagnosis, or as soon as possible during follow-up, about the increased risk of PD; however, patients should be advised that not all GD patients develop PD (<5%)	
	In order to align practice with the patient's preferences, it is important that GD patients receive the information on PD risk directly from their healthcare provider rather than being informed incidentally, without any appropriate counselling	
	The physician should adapt the communication of the PD risk to the social and cultural background of GD patients or parents of underage GD patients	
	Patients must feel free to ask their physician for information and to have all doubts and possible misunderstandings clarified. They need to be reassured that the physician will eventually provide the necessary support for an early identification of PD signs and symptoms and, in case of symptoms affecting the QOL, for an early therapeutic approach	
	The communication should state clearly that the lifetime risk to develop PD affects both patients and heterozygous carriers, with the GD patients having a higher risk and an earlier onset compared to the heterozygous carriers	
	At the time of GD diagnosis in a young underage patient, if the status of heterozygous is confirmed in the parents, information of an increased PD risk should be postponed to a later age, after transition from pediatric to an adult clinical setting and provided by the healthcare provider for adult patients	
**Management of PD risk in GD patients**	From the age of 35–40 years, every 12 months, healthcare providers should monitor for non-motor and motor symptoms or signs suggestive of PD	[11]
	The patient should be referred to neurologists when presenting with at least one clear motor sign alone or in the presence of any non-motor symptoms / signs with impact on the QOL	[15]

Table A11. Management goals for oral and dental manifestations in patients with GD1.

Category	Goal	Reference(s)
**Important considerations in the management of oral and dental manifestation in patients with GD**	Biopsy and bone grafting or dental extractions should not be performed exclusively because of concern about the abnormal appearance of the mandibular bony matrix on X-ray in the context of overwhelming Gaucher cells infiltration	[16]
	In cases where extractions have been performed consider implants in patients with GD using the same criteria as in other patients	
	The severity of thrombocytopenia does not predict the risk of bleeding even among patients receiving ERT; therefore, evaluation for coagulation deficiencies and impaired platelet function tests is prudent before commencing any dental procedures	
	Provide comprehensive follow up of the procedures to monitor possible complications; emphasize the importance of oral hygiene and appropriate dental and periodontal follow-up for all patients	
	Although dental involvement is a less common manifestation of GD, it is nonetheless imperative for dental practitioners to be aware of this disease, and to be familiar with the possible oral and dental complications that could develop	[6]

Table A12. Management goals for rare complications in patients with GD1.

Category	Goal	Reference(s)
**Gaucheroma**	Assess GD patients for Gaucheroma at baseline and ensure regular imaging is performed	[10]
	MRI/CT imaging is preferable in adults: ultrasound may be more practical in children	
	Monitor disease biomarkers for changes despite previously adequate disease modifying therapy	
	Involve hepatology (for liver Gaucheroma) and radiology specialists	
**GD lymphadenopathy**	MRI/CT imaging is preferable, if available	
	If using ultrasound, ensure the radiologist is experienced to reduce user-errors	
	Avoid biopsy	
	Surgical removal is not indicated	
	Dose frequency of ERT my need adjusting in severely affected patients	
	People with protein-losing enteropathy (PLE) should consume a diet rich in medium chain triglycerides and vitamin K supplements	
	People with PLE should be treated according to established guidelines	

Table A13.1 Treatment recommendations for GD1: Pharmacological intervention.

Category	Statement	Reference(s)
**General considerations**	Patients identified with GD mutations, who may be asymptomatic, do not require treatment at present, but must be monitored regularly (6-monthly) for disease progression according to the goals of treatment and indications to start therapy	[2]
	Detection of events that would prompt initiation of treatment in patients diagnosed with GD prior to the onset of signs or symptoms requires close monitoring (physical examination, biomarker assessment, complete blood count and determination of spleen and liver volumes every 6 -12 months)[*]	[4]
	Treatment should be initiated immediately in patients with GD1 who have significant and/or progressive disease	[4]
	Best outcomes are achieved by early initiation of therapy before onset of irreversible complications	[6]
	The need for lifelong therapy and the commitment of the time, money and effort should be discussed in detail with the patient and the caregivers prior to initiation of therapy to ensure compliance	
	Tailored treatment plan for each patient should be directed to symptom relief, general improvement of QOL, and prevention of irreversible damage	
	Bone Marrow Transplant is not the treatment of choice for GD1 due to the associated risk of complications and mortality	[14]

[*] It has been noted that these recommendations are for patients with overt manifestations and that more frequent monitoring of individuals without such findings may be warranted

Table A13.2 Treatment recommendations for GD1: First line therapy.

Category	Statement / Recommendation	Reference(s)
**Treatment initiation**	ERT is proven to be the standard of care in the therapeutic management of symptomatic patients with GD1	[6]
	There are currently three ERTs registered in South Africa: imiglucerase, velaglucerase alfa, and taliglucerase alfa. It is up to the treating physician to decide which ERT to use as there is no demonstration of superiority between the three available ERTs	[2]
	Early initiation of treatment is recommended with ERT as first-line treatment	[4]
	It is important to stress that patients may be asymptomatic, yet harbour significant disease manifestations such as cytopenia, splenomegaly and osteopenia; these patients should receive treatment to reverse disease manifestations	
	Maximal therapeutic gains can be achieved by pre-emptive therapy before irreversible complications occur compared to a ‘watchful waiting’ approach	[11]
**Benefits of treatment**	Early intervention with ERT in children with progressing and/or significant signs of GD may prevent the development of irreversible pathology	[4]
	Early administration of ERT has been shown to positively impact the growth of children	
	Therapy with ERT significantly ameliorates organomegaly and improves the hematological manifestations of GD	
	Treatment with ERT should be considered even if avascular osteonecrosis has occurred, as studies have shown that risk of further complications is reduced with ERT	[6]
	The therapeutic goals that can be attained with ERT should be discussed with the patient and the family	
**Pediatric patients**	Intravenous ERT is recommended for all children and adolescents with symptomatic GD1 and GD3 (i.e., any disease-related signs or symptoms)	[13]
**Pregnancy**	Continuation of ERT at the pre-pregnancy dose is not associated with any harm to the fetus or mother. Continuation of ERT may also decrease the risk for postpartum hemorrhage and the requirement for red blood cell transfusions	[4]

Table A13.3 Treatment recommendations for GD1: Dosage and administration of ERT.

Category	Statement / Recommendation	Reference(s)
**Dosage and administration of ERT**	The optimal dose of ERT needs to be individualized and will depend upon body weight and overall response to treatment. The weight of the patient is checked at each assessment and the dose adjusted according to the bodyweight	[5]
	Demonstration of a dose response to imiglucerase for hematological and visceral parameters and bone mineral density implies a need and provides a rationale for initial dose selection based on clinical severity	[2]
	Recommended starting dose for adult patients fulfilling indications for specific treatment but not fulfilling high-risk criteria is 15 units/kg (IV, over 2 hours) every other week (EOW)	[2]
	The recommended starting dose for children is 30 units/kg EOW	[2]
	For patients in whom immediate disease control is a priority, i.e., moderate to severe disease including those with life-threatening complications such as hepatopulmonary syndrome and pulmonary hypertension, the treating physician should consult with specialists and subject experts to titrate the patient's therapy according to clinical indicators	(6, 11)
	Recommended starting dose for severe or high-risk disease is 30 units/kg EOW	[2]
	There is insufficient evidence to support the widespread administration of ERT every four weeks	[13, 17]
	Dose may increase or decrease, based on achievement of therapeutic goals as assessed by routine comprehensive evaluations of the patient's clinical manifestations, followed by dose titration (up or down) according to achievement of therapeutic goals, and specific symptoms that are considered severe or higher risk	[2]
	Total dose to be administered at each infusion can also be partly adjusted based on the need to avoid wastage of the reconstituted drug as the drug is expensive and under no circumstances should be wasted	[6]
	If drug availability is limited, an initial dose of 30 units/kg may be used for less severely affected children but should be increased if treatment goals are not met	[6]
	Pre-infusion medication with antihistaminic / antipyretics is not routinely recommended unless there is history of infusion related reactions	[6]

Table A13.4. Treatment recommendations for GD1: Second line therapy

Category	Statement / Recommendation	Reference(s)
**Substrate reduction therapy (SRT)**	Miglustat is indicated for the oral treatment of adult patients with mild to moderate GD1, but only in those patients for whom ERT is unsuitable	[4]
	SRT is not recommended during pregnancy as there is a paucity of data on fetal outcomes for eliglustat	
	Eliglustat may be used as primary treatment in eligible adults with GD1	[4]
	CYP2D6 testing must be carried out prior to the use of eliglustat to determine eligibility and proper dosing	[4]
	Standard dosing of eliglustat: 84 mg twice daily (extensive / intermediate CYP2D6 metabolizer); 84 mg once daily (poor CYP2D6 metabolizer)	[18]
	Eliglustat is not recommended in patients who are pregnant / lactating; have pre-existing cardiac disease or long QT syndrome; in combination with Class IA, Class 1C and Class III antiarrhythmic medications; moderate to severe renal impairment, end-stage renal disease, dialysis, or prior kidney transplantation, hepatic impairment	[18]

Table A13.5. Treatment recommendations for GD1: Criteria for initiation of ERT, management of adverse events and indications for cessation of treatment

Category	Statement / Recommendation	Reference(s)
**Criteria for initiation ERT**	***ERT should be initiated in all symptomatic patients with one or more of the following features:***	
	Failure to thrive (height and weight less than the 5th percentile of age after excluding other causes) / growth failure / retardation / delayed puberty	[2, 6]
	Hepatosplenomegaly causing mechanical discomfort or splenic infarctions / history of progressive organomegaly	[2, 6]
	Severe cytopenia (Bicytopenia at least): hemoglobin <8 g/dL due to GD and not to other causes; platelets <60 x 10^9/L; leucocyte count <3 x 10^9/L	[2, 6]
	Symptomatic bone disease (bone pain, bone crisis), or active bone disease (osteopenia, fractures, marrow infiltration, infarction, osteonecrosis)	[6]
	Prior splenectomy (history of splenectomy is a marker for disease severity and such patients carry a high risk of avascular necrosis and osteonecrosis)	
	Symptomatic pulmonary involvement (evidence of pulmonary hypertension on 2D echocardiography, or evidence of Infiltrative lung disease on CT chest)	
	Impairment of function (physical and QOL)	[2]
	Any child with disease manifestations	
	There are no known drug interactions with ERTs; therefore, co-morbidities are not an indication to withhold therapy	
	It is important to discuss with the family and the patient the need to have life-long intravenous infusions every other week in hospital setting	[2, 6]
**Management of AE with ERTs**	Anaphylactic reactions have been reported in rare cases and necessary emergency medications must be available prior to infusion in cases at risk	[6]
	Management of infusion-related reactions include stopping the infusion temporarily and administration of antihistaminic and/or antipyretics. In such cases, the infusion can be restarted slowly after the infusion-related reaction settles down. Severe reactions may need management with steroids	

Category	Statement / Recommendation	Reference(s)
**Indications for cessation of treatment**	***Specific treatment may be withdrawn, following careful discussion with the patient and the multidisciplinary team, under the following circumstances:***	
	Intolerable and unavoidable adverse effects; intercurrent illness	[2]
	Where long-term QOL or expected survival is such that the patient will gain no significant benefit from specific treatment for GD	
	Lack of responsiveness to treatment, having made all appropriate dose adjustments and measures to improve effectiveness of treatment. This applies in the unlikely event of complete resistance to treatment and to irreversible progression of individual aspects of GD (most likely neurological) whereby the patient's QOL is very poor and where there is little or no prospect of response to treatment.	
	At the request of the patient, or properly allocated guardian acting in the patient's best interests, if the patient is properly deemed not competent	
	If the circumstances of the patient's lifestyle are such that sufficient compliance with treatment is not possible. Such cases might include intravenous drug abuse associated with a peripatetic lifestyle.	
	GD patients with an inadequate response to one ERT product may benefit from a switch to an alternative agent in the class, or to an SRT	[4]

Table A14. Supportive Management of GD1.

Category	Statement / Recommendation	Reference(s)
**Adjuvant therapy**	Supportive management is an extremely important part of management of patients with GD, especially children affected with the disease. It includes correction of nutritional anemia, vitamin D deficiency and improvement of the nutritional status of the patient	[6]
	Supportive therapy is indicated for those patients who decline the above option (usually elderly patients) and require symptomatic supportive intervention with blood products, bisphosphonate therapy, and/or analgesia	[2]
	Dependency on blood transfusions for severe anemia is a marker of severe end-stage disease: each blood transfusion rarely lasts more that 2-3 weeks and transfused blood cells massively add to the load of glycolipids leading to rapid acceleration of the disease	[6]
	Dependency on blood transfusion is an urgent indication for ERT with the goal to eliminate the need for blood transfusion	
	Immunization with vaccines as per the National guidelines and the recommended immunization schedule for splenectomized patients should be followed	
	Withholding splenectomy if therapy with ERT / SRT is feasible in a particular patient, unless it is required as a life-saving measure	
	Adult patients may benefit from bisphosphonate therapy either orally or intravenously. Guidelines for the use of bisphosphonates should be similar to those of other causes of osteopenia / osteoporosis	[2]
	Other treatment considerations should include Vitamin D, calcium, specific pain medication, seizure/neurological management, and pulmonary hypertension management	[2]
**Surgical management**	Orthopedic surgical intervention is commonly required to restore function and correct deformity. Subchondral bone collapse as a result of avascular necrosis leads to pain and loss of function and may require joint replacement, most commonly of the hip joint, but also of the knee and shoulder. Given that joint replacement is often carried out at a young age, there is frequent requirement for complex revision surgery	[2]
	Gallstone disease is also more prevalent in GD	[2]
	Peri-operative medical management of GD patients must take account of: (i) the risk of infection in the asplenic patient; and (ii) the risk of bleeding associated with thrombocytopenia, platelet function defects and coagulopathies associated with the condition	[2]
**Other**	Mobility aids, e.g., crutches and wheelchairs should be provided to patients who need it to aid mobility for everyday living	[2]

Amended statements

Table A15. Statements that needed amendment before consensus was reached

Category	Statement	Reference	Outcome
**Lung infiltration**	**Original statement:** Mucolytics, guided by a respiratory team, are recommended for patients with decreased FVC, Dlco <80%, severe chest infections and/or recurrent hospital admissions due to chest infections<br><br>**Amended statement:** *Patients with decreased FVC, DLCO <80%, severe chest infections and/or recurrent hospital admissions due to chest infections should be referred to a pulmonologist to guide further management*	[10]	After round one, consensus on this statement was 60%. It was rephrased for the second round of voting, during which consensus was achieved.
**General disease management**	**Original statement:** Monitoring treated patients with Lyso-Gb1 in addition to the usual standard of care for predicting long-term clinical outcomes in patients with GD<br><br>**Amended statement:** *Monitoring treated patients with Lyso-Gb1/Lyso-GL1 in addition to the usual standard of care for predicting long-term clinical outcomes in patients with GD*	[4]	After round one, consensus on this statement was 60%. It was rephrased for the second round of voting, during which consensus was achieved.
**First line therapy: treatment initiation**	**Original statement:** In South Africa, imiglucerase is the treatment of choice / SOC for GD1<br><br>**Amended statement:** *There are currently three ERTs registered in South Africa: imiglucerase, velaglucerase alfa, and taliglucerase alfa. It is up to the treating physician to decide which ERT to use as there is no demonstration of superiority between the three available ERTs*	[2]	After round one, consensus on the original statement was 60%. Consensus could still not be reached during round 2 discussions and the panel agreed to independently review literature on the subject and revote during round 3. During round 3 discussions, the statement was rephrased through a collaborative process by all panelists.
**Dosage and administration of ERT**	**Original statement:** Recommended starting dose for adult patients fulfilling indications for specific treatment but not fulfilling high-risk criteria is 10 – 30 U/kg (IV, over 2 hours) every other week (EOW)<br><br>**Amended statement:** *Recommended starting dose for adult patients fulfilling indications for specific treatment but not fulfilling high-risk criteria is 15 U/kg (IV, over 2 hours) every other week (EOW)*	[2]	Although consensus on the original statement was 80% after this first round of voting, after discussions in round 3, panel members recommended removing dosage ranges for statements regarding recommended dosages of ERT
**Dosage and administration of ERT**	**Original statement:** The recommended starting dose for children is 30–60 U/kg every other week (EOW)	[2]	Although consensus on the original statement was 80% after this first round of voting, after discussions in round 3, panel members recommended removing

**Category**	**Statement**	**Reference**	**Outcome**
	**Amended statement:** *The recommended starting dose for children is 30 U/kg every other week (EOW)*		dosage ranges for statements regarding recommended dosages of ERT
**Dosage and administration of ERT**	**Original statement:** Recommended starting dose for severe or high-risk disease is 30 – 60 U/kg EOW<br><br>**Amended statement:** *Recommended starting dose for severe or high-risk disease is 30 U/kg EOW*	[2]	Although consensus on the original statement was 80% after this first round of voting, after discussions in round 3, panel members recommended removing dosage ranges for statements regarding recommended dosages of ERT
**Dosage and administration of ERT**	**Original statement:** For patients in whom immediate disease control is a priority, i.e., moderate to severe disease including those with life-threatening complications such as hepatopulmonary syndrome and pulmonary hypertension, the initial dose of imiglucerase is usually 120 U/kg body weight in a four-week period<br><br>**Amended statement:** *For patients in whom immediate disease control is a priority, i.e., moderate to severe disease including those with life-threatening complications such as hepatopulmonary syndrome and pulmonary hypertension, the treating physician should consult with specialists and subject experts to titrate the patient's therapy according to clinical indicators.*	[6, 11]	After round one, consensus on the original statement was 60%. Consensus could still not be reached during round 2 discussions and the panel agreed to independently review literature on the subject and revote during round 3. During round 3 discussions, the statement was rephrased through a collaborative process by all panelists.

B. Statements where consensus could not be reached

Table A16. Statements for which consensus could not be reached

Category	Statement	Reference	Outcome
**Lung infiltration**	Lung transplantation may be considered in patients with end stage lung disease secondary to GD	[10]	After round one, consensus on this statement was 60%. During round 2 discussions, consensus was that the statement should be removed.
**Pulmonary hypertension (PH)**	Consider lung transplantation for patients with end-stage GD1-related PH	[10]	After round one, consensus on this statement was 60%. During round 2 discussions, consensus was that the statement should be removed.
**Follow-up imaging**	Heel ultrasound if indicated to assess bone involvement if evaluation at other sites not possible by DEXA	[2]	After round one, consensus on this statement was 40%. During round 2 discussions, consensus was that the statement should be removed.
**Communication of PD risk in GD patients**	The patient has the right to refuse to be informed on this risk	[15]	After round one, consensus on this statement was 60%. During round 2 discussions, consensus was that the statement should be removed.
**Indications for cessation of treatment**	If the health and well-being of medical and/or nursing staff are placed under significant threat as a result of the actions or lifestyle of the patient	[2]	After round one, consensus on this statement was 60%. During round 2 discussions, consensus was that the statement should be removed.
**Dosage and administration of ERT**	Standard dose of ERT for adult patients with less severe disease at initiation is 60 units per kg (U/kg) bodyweight every 4 weeks	[6, 11]	After round one, consensus on this statement was 40%. Consensus could still not be reached during round 2 discussions and the panel agreed to independently review literature on the subject and revote during round 3. During round 3 discussions, the consensus was to remove this statement and provide a more comprehensive discussion on recommended dosing
**Dosage and administration of ERT**	The recommendation according to the results with ERT in clinical trials is to start at dose 60 units/kg every other week, until therapeutic goals are achieved and maintain doses with progressive reduction without dropping below 15 units/kg every two weeks	[13, 17]	
**Current SOC**	The current first line treatment for GD1 in South Africa for adult GD1 patients with mild disease is: **Imiglucerase, 10 – 30 U/kg (IV, over 2 hours) every other week (EOW)**	[2]	After round one, consensus on this statement was 40%. Consensus could still not be reached during round 2 discussions and the panel

**Category**	**Statement**	**Reference**	**Outcome**
**Current SOC**	The current first line for GD1 in South Africa for GD1 adult patients with moderate to severe, or high-risk disease is: **Imiglucerase, 30 – 60 U/kg every other week (EOW)**	[2]	agreed to independently review literature on the subject and revote during round 3. During round 3 discussions, the consensus was to remove this statement and provide a more comprehensive discussion on the recommendations on SOC
**Current SOC**	The current first line for GD1 in South Africa for pediatric GD1 patients is: **Imiglucerase, 30–60 U/kg every other week (EOW)**	[2]	
**Current SOC**	The current second line treatment for GD1 in South Africa for adult patients is: **Miglustat, 100mg three times daily (TDS)**	[19]	
**Future SOC**	Considering all the treatment options available for the treatment of GD1, the SOC for South African patients with GD1 should be ERT at a standard dose of 60 **U/kg every other week, individualized according to response and requirements**	[20, 21]	

REFERENCES

1. Pastores GM, Weinreb NJ, Aerts H, Andria G, Cox TM, Giralt M, et al. Therapeutic goals in the treatment of Gaucher disease. Semin Hematol. 2004;41(4 Suppl 5):4-14.

2. Bhengu L, Davidson A, du Toit P, Gerntholtz T, Govendragaloo K, Heitner R, et al. South African guidelines for the management of Gaucher disease, 2011. S Afr Med J. 2012;102(8):697-702.

3. Biegstraaten M, Cox TM, Belmatoug N, Berger MG, Collin-Histed T, Vom Dahl S, et al. Management goals for type 1 Gaucher disease: An expert consensus document from the European working group on Gaucher disease. Blood Cells Mol Dis. 2018;68:203-8.

4. Kishnani PS, Al-Hertani W, Balwani M, Göker-Alpan Ö, Lau HA, Wasserstein M, et al. Screening, patient identification, evaluation, and treatment in patients with Gaucher disease: Results from a Delphi consensus. Mol Genet Metab. 2022;135(2):154-62.

5. Linari S, Castaman G. Clinical manifestations and management of Gaucher disease. Clin Cases Miner Bone Metab. 2015;12(2):157-64.

6. Puri RD, Kapoor S, Kishnani PS, Dalal A, Gupta N, Muranjan M, et al. Diagnosis and Management of Gaucher Disease in India - Consensus Guidelines of the Gaucher Disease Task Force of the Society for Indian Academy of Medical Genetics and the Indian Academy of Pediatrics. Indian Pediatr. 2018;55(2):143-53.

7. Giraldo P, Andrade-Campos M. Novel Management and Screening Approaches for Haematological Complications of Gaucher's Disease. J Blood Med. 2021;12:1045-56.

8. Giuffrida G, Cappellini MD, Carubbi F, Di Rocco M, Iolascon G. Management of bone disease in Gaucher disease type 1: clinical practice. Adv Ther. 2014;31(12):1197-212.

9. Hughes D, Mikosch P, Belmatoug N, Carubbi F, Cox T, Goker-Alpan O, et al. Gaucher Disease in Bone: From Pathophysiology to Practice. J Bone Miner Res. 2019;34(6):996-1013.

10. Ramaswami U, Mengel E, Berrah A, AlSayed M, Broomfield A, Donald A, et al. Throwing a spotlight on under-recognized manifestations of Gaucher disease: Pulmonary involvement, lymphadenopathy and Gaucheroma. Mol Genet Metab. 2021;133(4):335-44.

11. Mistry PK, Cappellini MD, Lukina E, Ozsan H, Mach Pascual S, Rosenbaum H, et al. A reappraisal of Gaucher disease-diagnosis and disease management algorithms. Am J Hematol. 2011;86(1):110-5.

12. Zion YC, Pappadopulos E, Wajnrajch M, Rosenbaum H. Rethinking fatigue in Gaucher disease. Orphanet J Rare Dis. 2016;11(1):53.

13. Weinreb NJ, Goker-Alpan O, Kishnani PS, Longo N, Burrow TA, Bernat JA, et al. The diagnosis and management of Gaucher disease in pediatric patients: Where do we go from here? Mol Genet Metab. 2022.

14. Gary SE, Ryan E, Steward AM, Sidransky E. Recent advances in the diagnosis and management of Gaucher disease. Expert Rev Endocrinol Metab. 2018;13(2):107-18.

15. Di Rocco M, Di Fonzo A, Barbato A, Cappellini MD, Carubbi F, Giona F, et al. Parkinson's disease in Gaucher disease patients: what's changing in the counseling and management of patients and their relatives? Orphanet J Rare Dis. 2020;15(1):262.

16. Saranjam HR, Sidransky E, Levine WZ, Zimran A, Elstein D. Mandibular and dental manifestations of Gaucher disease. Oral Dis. 2012;18(5):421-9.

17. Shemesh E, Deroma L, Bembi B, Deegan P, Hollak C, Weinreb NJ, et al. Enzyme replacement and substrate reduction therapy for Gaucher disease. Cochrane Database Syst Rev. 2015;2015(3):Cd010324.

18. Balwani M, Burrow TA, Charrow J, Goker-Alpan O, Kaplan P, Kishnani PS, et al. Recommendations for the use of eliglustat in the treatment of adults with Gaucher disease type 1 in the United States. Mol Genet Metab. 2016;117(2):95-103.

19. Zavesca [package insert on the Internet]. Midrand: Janssen Pharmaceutica Pty Ltd; 2021 [cited 2022 May 23]. Available from: https://pi-pil-repository.sahpra.org.za/wp-content/uploads/2021/11/Zavesca-Final-Approved-PI_12-October-2021.pdf.

20. Bennett LL, Fellner C. Pharmacotherapy of Gaucher Disease: Current and Future Options. P t. 2018;43(5):274-309.

21. Shawky RM, Elsayed SM. Treatment options for patients with Gaucher disease. Egyptian Journal of Medical Human Genetics. 2016;17(3):281-5.
